# Grass–Endophyte Interactions and Their Associated Alkaloids as a Potential Management Strategy for Plant Parasitic Nematodes

**DOI:** 10.3390/toxins16060274

**Published:** 2024-06-15

**Authors:** Nyambura G. Mwangi, Mark Stevens, Alistair J. D. Wright, Simon G. Edwards, Martin C. Hare, Matthew A. Back

**Affiliations:** 1 Agriculture and Environment Department, Harper Adams University, Newport TF10 8NB, UK; sedwards@harper-adams.ac.uk (S.G.E.); mhare@harper-adams.ac.uk (M.C.H.); mback@harper-adams.ac.uk (M.A.B.); 2 British Beet Research Organisation, Centrum, Norwich Research Park, Colney Lane, Norwich NR4 7UG, UK; mark.stevens@bbro.co.uk (M.S.); alistair.wright@bbro.co.uk (A.J.D.W.)

**Keywords:** endophyte, *Epichloë*, mechanisms, alkaloids, induced resistance

## Abstract

Claviceptaceous endophytic fungi in the genus *Epichloë* mostly form a symbiotic relationship with cool-season grasses. *Epichloë* spp. are capable of producing bioactive alkaloids such as peramines, lolines, ergot alkaloids, and indole-diterpenes, which protect the host plant from herbivory by animals, insects, and nematodes. The host also benefits from enhanced tolerance to abiotic stresses, such as salt, drought, waterlogging, cold, heavy metals, and low nitrogen stress. The bioactive alkaloids produced can have both direct and indirect effects towards plant parasitic nematodes. Direct interaction with nematodes’ motile stages can cause paralysis (nematostatic effect) or death (nematicidal effect). Indirectly, the metabolites may induce host immunity which inhibits feeding and subsequent nematode development. This review highlights the different mechanisms through which this interaction and the metabolites produced have been explored in the suppression of plant parasitic nematodes and also how the specific interactions between different grass genotypes and endophyte strains result in variable suppression of different nematode species. An understanding of the different grass–endophyte interactions and their successes and failures in suppressing various nematode species is essential to enable the proper selection of grass–endophyte combinations to identify the alkaloids produced, concentrations required, and determine which nematodes are sensitive to which specific alkaloids.

## 1. Introduction

Cool-season grasses (family Poaceae) are often associated with claviceptaceous, endophytic fungi in the genus *Epichloë* [1,2,3,4,5,6,7]; around 30% of Poaceae species have a relationship with *Epichloë* species [4,8]. Over 600 species from this family are used for foraging and originate from Europe, Africa, and western Asia [9]. Meadows and pastures cover approximately 30% of the agricultural land in Europe [10]. Forage grasses are critical to livestock operations and can be native or introduced, perennial or annual, cool-season or warm-season, depending on the region and farmer preference [9]. *Epichloë* endophytes enhance host grasses’ tolerance to biotic and abiotic stresses such as salt, drought, waterlogging, cold, heavy metals, and low nitrogen stress, hence improving their adaptation to grassland ecosystems [9,11,12,13,14,15]. The host also benefits through increased uptake of nutrients and production of metabolites that shield it against parasites and herbivores [4,8]. Over the years, *Epichloë* endophytes have been associated with fescue toxicosis in livestock which negatively impacts animal productivity leading to economic losses. Toxicity to livestock occurs because the endophytes can produce indole-diterpene alkaloids and ergot alkaloids in pasture grasses [16,17,18]. Grass breeders have identified novel strains of fungal endophytes with low toxicity to livestock [7,19,20] and good activity towards insect pests [11,12]. These include NEA2 and AR1, which do not produce lolitrem B and strains AR37 and NEA12, which produce epoxy-janththitrems, a group of indole-diterpenes with structural similarities to lolitrem B [21,22]. A good example is also the interaction between the fungal endophyte *Epichloë uncinata* and the meadow fescue *(Festuca pratensis*), where the loline alkaloids produced have been demonstrated to effectively manage insects such as the root aphid, pasture mealy bug, black beetle, grass grub, and Argentine stem weevil in New Zealand by acting as feeding deterrents [23]; most importantly, loline alkaloids do not cause animal health disorders (fescue toxicosis and ryegrass staggers) as is the case with other alkaloids [20,24]. This review focuses on the potential of grass–endophyte interactions in the suppression of various plant parasitic nematodes in plants by either limiting nematode multiplication or directly by causing paralysis, mortality, and reduced hatching of nematode eggs through the release of toxic metabolites. There are striking examples of these interactions conferring resistance to plant parasitic nematodes and secondary metabolites/alkaloids produced by the endophytes directly interacting with the nematodes.

## 2. Grass–Endophyte Interactions

Fungal species within the genus *Epichloë* are obligate symbionts and are transmitted both vertically and horizontally, i.e., via seeds and spores, respectively [9]. The genera previously classified as *Acremonium* and *Neotyphodium* were synonymised under the genus *Epichloë* [9,14,25]. The fungal hyphae grow between the cells of the leaf tissue and attach to the cell wall of the plants where they absorb nutrients. Unlike other fungi, the cells grow via intercalary expansion rather than apical growth [14,18]. The hyphal growth is synchronized with the host grasses, with growth beginning when the leaves and other tissues are being formed and ceasing when the surrounding tissue has matured. The hyphae eventually colonise the new seed when the host reaches maturity; therefore, the endophyte is transferred to the next generation [4]. The prevalence of the mutualism between grasses and endophytes is attributed to a defensive mutualism hypothesis, suggesting that the fungi help defend the plants against herbivores through the production of toxic alkaloids [9]. The mechanisms that are involved in a successful mutualism have not been fully explored, as plants typically elicit defence responses such as hypersensitivity when they are invaded by foreign organisms [26]. The absence of the defence response by the grass species upon colonisation by the endophyte suggest a complex relationship that might involve communication between the two species [27]. However, the mutualism is not always successful, as certain species of *Epichloë* exhibit pleiotropic behaviour, causing choke disease in some tillers while remaining symptomless in others. Some species of *Epichloë*, such as *E. amarillans*, *E. brachyelytri*, *E. elymi*, and *E. festucae*, have this pleiotropic condition [18]. Several mechanisms involved in a successful mutualistic interaction have been proposed by various researchers. For example, the proposition that the fungus produces an inhibitor which suppresses recognition by papain-like cysteine proteases, that are involved in eliciting plant immune responses to pathogens [28]. The endophyte has also been reported to counter ROS (reactive oxygen species) production, by producing NoxA which is a hydrolase enzyme that regulates superoxide production, known to inhibit fungal growth in perennial ryegrasses [29]. *Epichloë* endophytes’ protective mechanisms in grass hosts include the production of harmful alkaloids, increased antioxidant production, changes in gene expression, and the creation of other substances with physiological effects [9]. The co-evolution of endophytes and grasses has transformed their relationship from pathogenic to mutualistic, with antioxidants playing a significant role in stress defence [9]. The relationship between *Epichloë* endophytes and plant hosts is very specific, leading to the production of different alkaloids which sometimes cannot be produced by either species alone. The alkaloid profiles vary depending on the host–endophyte combinations [17]. Some of the common and most studied relationships between *Epichloë* endophytes and host grasses include the following: *E. coenophialum*–*Festuca arundinacea* interaction, *E. festucae* var. lolii–*Lolium perenne* interaction, and *E. gansuensis*–*Achnatherum inebrians* interaction [9]. *Festulolium* hybrids are intergeneric crosses between *Festuca pratensis* and *Lolium perenne* and/or *Lolium multiflorum*. *Epichloë uncinata* colonises these hybrids [1]*. Epichloë* spp. are capable of producing bioactive alkaloids such as peramines, lolines, ergot alkaloids, and indole-diterpenes which protect the host plant from herbivory by animals, insects, and nematodes [1,3,19,30,31]. Endophyte–grass relationships also stimulate the production of phenolic compounds and flavonoids, which can alter the soil microbiome. The relationship also stimulates the production of amino acids and sugars under stress, assisting in osmoregulation and nutrient availability. Certain grasses colonised by endophytes are allelopathic to other species due to the release of toxins in exudates [9]. While grasses can produce allelopathic chemicals as individuals, they can also produce allelopathic chemicals in groups. Endophytes living in above-ground parts of the plant can increase the exudation of phenolic compounds and other metabolites from the roots. However, these compounds are not always associated with endophyte presence; for instance, chlorogenic acid, caffeoylquinic acid isomers, and 3,5-DICQA have been isolated in the roots of both E+ and E− plants [32]; however, the amount of carbohydrates and organic carbon phenolic compounds released by E+ grass has been shown to be greater than E− grasses [33]. The grass–endophyte interaction has also been shown to have an effect on the compounds exuded, hence influencing the soil microbiome. [9]. The root exudates of grasses colonised with *Epichloë* were found to promote the growth and colonization of roots in *Bromus auleticus*, a potential forage grass, by arbuscular mycorrhizal fungi as well as by dark septate endophytes. Endophyte-colonised tall fescue microbiomes had, in general, a very diverse fungal population at the genus level. Fungi associated with E− and E+ also showed a significant shift from basidiomycetes to ascomycetes, respectively [34].

## 3. Secondary Metabolites Associated with Grass–Endophyte Interactions

The major classes of secondary metabolites associated with grass–endophyte interactions, include various alkaloids such as ergot alkaloids, aminopyrrolizidine alkaloids, the pyrolopyrazine alkaloids, the indole diterpenoid alkaloids, and the 11,12-epoxy-janthitrems [18]. Pyrrolizidine alkaloids are found in about 3% of flowering plants and are a significant source of plant toxins. These pyrrolizidine unsaturated alkaloids are esters of hydroxylated 1-methylpyrolizidine [30]. However, the saturated amino pyrrolizidines are the most important in the graminae species [35]. In tall fescue and Lolium–Festuca hybrids, the presence of a fungal endophyte in the plant tissue is required for the significant accumulation of pyrrolizidine alkaloids, including loline [1]. *Epichloë* spp. produce four types of alkaloids, namely, lolines, indole-diterpenes, peramines, and ergot alkaloids [9]. Phenolic compounds, antioxidants, and reactive oxygen species are also produced in the mutualism. It is well-known that both grasses and endophytes can alter their own metabolic pathways during their mutualistic relationship to produce different alkaloid metabolites [9]. Complex pathways involving specific gene clusters are used to create various alkaloids. However, there is evidence of crosstalk and plant defence responses in the mutualistic relationship between *Epichloë* spp. and cool-season grasses. Alkaloids are found to occur differently in various grass–endophyte combinations and may also be influenced by host genotypes, where they may all be present or some may be absent [18,36]. The pyrolopyrazine alkaloids such as peramine occur in the majority of grass–endophyte interactions [7], followed by ergot alkaloids (ergovaline), lolines (aminopyrrolizidine alkaloid), and lastly by lolitrem (indole diterpenoid alkaloid) [18]. The plant genotype and endophyte strain are the major determinants of the type of alkaloid produced [36,37,38].

### 3.1. Lolines

Lolines were first isolated from the seeds of *Lolium cuneatum*. Lolines and their derivatives have a unique structure, where they contain an oxygen bridge between carbon 2 and 7. They are water soluble and able to translocate around host tissues to areas such as the roots, where the endophyte itself is not found actively growing [39]. Only a small percentage of grass species with claviceptaceous endophytes have lolines, according to surveys, when compared to other alkaloids, e.g., ergot alkaloids which are widely distributed. However, lolines are found in higher concentrations than other alkaloids when they occur [7]. Different grass species and accessions have different levels and types of lolines. Two genes, *lolA* and *lolC*, were discovered in all loline-alkaloid-producing endophytes but absent from non-producers [7]. The type of loline alkaloids produced by colonised grasses include norloline (NL), loline (L), N-methylloline (NML), N-formylnorloline (NFNL), N-acetylnorloline (NANL), N-formylloline (NFL), and N-acetylloline (NAL). NFL and NAL are the most commonly isolated loline alkaloids from Lolium–Festuca hybrids [1]. The endophyte’s presence is required for high loline alkaloid expression and the fungus genotype determines whether lolines are produced or not [40], and in tall fescue, loline production was positively associated with the presence of the endophytic fungus *E. typhina* [41]. The fungal source of lolines was also confirmed in devised culture conditions that induced loline alkaloid production by *E. uncinatum*, an endophyte associated with extremely high levels of lolines (up to 20 mg/g plant dry weight), in meadow fescue [40]. *Epichloë uncinata* produces loline alkaloids, which can accumulate to make up 2% of the host plant dry weight [1,42]. The concentration of the alkaloids is higher in the seed than in the vegetative tissues. In the vegetative tissue, the alkaloids are highest in the spikelet and stem compared to the leaf blade [38]. Since the endophyte is absent in the roots, the presence of the alkaloids has been attributed to translocation in the xylem [1].

### 3.2. Ergot Alkaloids

Ergot alkaloids are secondary metabolites produced by certain fungi, e.g., *Claviceps purpurea*, that have diverse effects on various organisms. Metabolites in the ergot family are classified as clavines, simple amides of lysergic acid or ergopeptines based on their relative position in the pathway or their complexity [43]. Ergot alkaloids are characterised by the presence of an ergolene ring system, and the majority are derivatives of lysergic acid. Lysergic acid amine and ergovaline are the most predominant members of ergot alkaloids [7]. Other ergopeptines produced by *Claviceps* include ergotamine, ergosine, ergocornine, ergocryptine, and ergocristine [44]. Intermediates and spur products are also produced in the ergot alkaloid synthesis system and are also beneficial to the endophyte–grass relationship [45]. The activities of clavine intermediates and spur products compared to ergopeptines or simple amides have been reported to differ in direct exposure studies to nematodes and bacteria [32,45]. This was shown to be true in a study with perennial ryegrass (*Lolium perenne*) and a knockout mutant of the endophyte *Epichloë* typhina × *Epichloë lolii* isolate Lp1 that accumulates certain clavines but not ergopeptines or simple amides of lysergic acid. This endophyte had lower insecticidal and insect feeding deterrent properties in comparison to a wild-type endophyte, indicating that ergopeptines and simple amides of lysergic acid have a crucial role in insect defence [46]. Ergot alkaloids are known to have negative effects on mammals. Ergovaline is mostly known to be toxic to grazing mammals; it interferes with the neurotransmitter receptors, causing muscle contraction, alterations in the nervous and reproductive systems, and also vasoconstriction. They act on the monoamine neurotransmitters, serotonin, dopamine, adrenaline, and noradrenaline receptors. Organisms such as insects and nematodes that possess homologous neurotransmitters are also affected. Some of the effects on insects include increased mortality, feeding deterrence, and delayed development [46].

### 3.3. Indole-Diterpenes

Like the ergot alkaloids, indole-diterpenes are another class of very diverse alkaloids. Certain *Epichloë* spp., *Claviceps* spp., and some members of Tricho-comaceae (e.g., *Aspergillus* and *Penicillium* spp.) produce indole-diterpenes [47]. The indole-diterpene alkaloids include tremorgenic neurotoxins commonly known as lolitrems. Lolitrem B is the most abundant, produced by *E. festucae* var. lolii in symbiotic association with perennial ryegrass. It is also the most potent of the indole-diterpenes and is associated with “ryegrass staggers” disease of sheep [48]. Recent genetic screening, aided by a better understanding of indole-diterpene biosynthesis, revealed that some epichloid endophytes that do not produce lolitrem B still produce simpler indole-diterpenes such as terpendoles [7]. Endophytes producing terpendoles without lolitrem B are less toxic alternatives to traditional perennial ryegrass varieties but may still cause minor shaking in mammals due to the presence of janthitrems or other indole-diterpenes [49]. The less common but biogenically related janthitrems may also have insecticidal activity. Janthitrems accumulate in plants with *N. lolii* isolate AR37, an endophyte species that is included in some commercial varieties of perennial ryegrass due to its low tremorgenic activity. AR37-colonised perennial ryegrass varieties are particularly resistant to the porina moth (*Wiseana cervinata*) [50]; however, no direct link between AR37’s anti-insect activities and the janthitrems has been established. Other indole-diterpenes have been isolated from the sclerotia of various *Aspergillus* spp. and these indole-diterpenes have been shown to have anti-insect properties through feeding and topical tests [43].

### 3.4. Peramine

Peramine is the most commonly distributed pyrrolopyrazine alkaloid in nature [43]. A single gene (*perA*) was identified to be responsible for the biosynthesis of peramine in grass–endophyte symbiosis; knockout of the same gene led to inhibition of peramine production and loss of resistance to the Argentine stem weevil (*Listronotus bonariensis*) [51]. Peramine is derived from a dipeptide made up of arginine and a precursor to proline, and is unique among the four major alkaloids in that it is a single chemical as opposed to a family of chemicals, while the biosynthetic pathway involves a single multifunctional enzyme unlike other alkaloids [43]. Peramine is water soluble and is distributed throughout the plant unlike other alkaloids which are localised in different plant tissues [52]. It is known for being a feeding deterrent against the Argentine stem weevil, a common pest of perennial ryegrass, as well as other insects [38]. Its presence in the roots, where the endophyte does not colonise, indicates that it translocated to the roots. Peramine has also been found in fluids of cut leaves of endophyte-colonised grasses; this location is essential in deterring sensitive insect pests from damaging the plant cuticle [43]. Table 1 highlights the occurrence and concentrations of the different alkaloids in the different grass–endophyte combinations.

## 4. Distribution and Concentration of Alkaloids in Plant Tissues

Alkaloids in grass–endophyte interactions are distributed variably in the different plant parts, with the roots, shoots, and seeds all containing alkaloids in varying amounts. When compared to vegetative tissue, mature seeds have a higher alkaloid content [22]. In the case of the occurrence of loline alkaloids in vegetative tissues, pseudostems have higher levels of N-acetylloline (NAL) and N-formylloline (NFL) than the leaf blade or sheath of a culm [55]. Loline alkaloids also occur in the root system, which suggests that they are transferred from the leaf sheath and stem, which are thought to be synthesis sites, and translocated through the phloem and possibly upwards in the xylem [55]. Endophytic fungal hyphae in plants follow a gradient with high concentrations in the basal regions to low concentrations at the apical parts, and colonise grasses exclusively above ground [1,7,56]. The association’s highly compatible nature is responsible for this carefully controlled growth. Although the hyphae located in old, mature host tissues are metabolically active, the growth of endophytic hyphae is linked to the host’s life cycle [38]. Peramine alkaloid is found primarily in the shoots and occurs in very low levels in the roots, whereas ergovaline is found to be distributed throughout the tissues, with a 26 percent reduction in the roots [22,53]. The alkaloid–endophyte ratios in grass tissues have distinct distributions depending on the type of alkaloid and grass genotype. For instance, the ergovaline–endophyte ratio is higher in the basal plant tissues, while for lolitrem B and peramine, the ratio is higher in the apical tissue [38]. The distribution of intermediate alkaloids in the indole-diterpene pathway, which leads to the production of lolitrem B and epoxy-janthitrem I, varied in concentration between the shoots and roots, with the roots showing a decrease. Furthermore, paxilline and epoxy-janthitrem I were found to have a more even distribution between the shoots and roots than other alkaloids [22]. Both the grass and fungal species influence the alkaloid profile in grass–endophyte interactions. *Festuca rubra* colonised with *E. festucae* had a significantly high concentration of ergovaline compared to other *Festuca* spp.; however, the peramine alkaloid was not found and ergovaline accumulation was found to be greater in the leaf sheath [7]. Age has also been shown to influence the alkaloid–endophyte ratio in some alkaloids like lolitrem B, which increases with age, although this trend was not observed for other alkaloids, highlighting the greater stability of lolitrem B in the plant. The study showed that endophyte colonisation had a minor influence on the alkaloid concentration and that alkaloid–endophyte ratios were more affected by host genotype and this was specific for each alkaloid [38]. Until senescence, peramine, ergovaline, and lolitrem B are produced, with peramine decreasing while lolitrem B remains high [7]. Wounding of plants has also been reported to markedly influence the concentration of loline alkaloids. The loline concentration in meadow fescue colonised with *Neotyphodium siegelii* increased from 0.1% to 1.9% of plant dry mass from 0 to 11 days post-clipping [16]. A similar result was also obtained when tall fescue colonised with *E. coenophialum* was artificially damaged; the loline concentration in the damaged plants increased nearly two fold for E+ plants (1.16%) compared to the control undamaged E+ plants (0.63%) [57]. The concentration of alkaloids is also known to be influenced by environmental factors such as temperature, season, humidity, and nutrient levels. In tall fescue colonised with *E. coenophialum*, the concentration of ergot alkaloids in shoots increased with increases in phosphorus availability from 17 to 50 mg kg^−1^ but declined again at 96 mg kg^−1^ soil. However, the concentration in the roots increased linearly with increasing soil phosphorus [13]. Temperature is another important factor affecting the concentration of endophyte alkaloids. In the early spring, ergovaline and lolitrem B levels are usually low, but they rise with rising temperatures and reproductive development before falling off in post-reproductive regrowth. Concentrations rise again in the summer due to water stress, temperature, and the accumulation of older leaves. Sun-cured hay samples yielded around 80 percent of the starting alkaloids; NAL and NFL are stable in dehydrated hay as no changes were observed in hay samples kept in the laboratory for over 15 years [9,55]. Ergovaline and lolitrem B remain in senescent and dead leaves, making them potential sources of toxicants in pasture, hay, and silage [20]. Tall fescue showed a gradient in genetic composition, with different clusters found within various geographical locations. According to the study, *E. uncinata* strains in S*. pratensis* subpopulations seem to follow a geographic pattern rather than an association with a specific host genotype. Endophyte subpopulations showed significant differences in loline production, with Eu_P3 having the highest average expression of lolines. Increasing levels of total lolines were driven by increasing levels of NFL, with minor contributions from NANL and NAL [58]. Despite the fact that the endophyte *E. uncinata* does not require a specific host genotype to survive, it exerts significant effects on alkaloid levels and the fungal mycelial biomass. Endophyte–host compatibility is found to be independent of geographic and host genetic distances, implying that *E. uncinata* has a certain plasticity in colonising different genotypes of *S. pratensis* [58]. This is different for *E. festucae* where the level of alkaloid production was reported to be influenced more by the plant genotype than the endophyte genotype.

## 5. Direct Effects of Secondary Metabolites to Nematodes

Secondary metabolites produced as a result of grass–endophyte interactions can have both direct and indirect effects to plant parasitic nematodes. Directly, they can interact with the nematodes’ motile stages causing paralysis (nematostatic) or death (nematicidal) [56]. Direct interaction of the secondary metabolites with soil-inhabiting plant parasitic nematodes involves the translocation of the compounds to the root systems and subsequent exudation, which then has a negative effect on the development and reproduction of the nematode [59]. The metabolites can also interact with immobile stages such as nematode eggs causing hatching inhibition [60]. To investigate the direct effects of these metabolites, nematodes are exposed to the metabolites in an in vitro bioassay. This involves exposing the nematodes to biologically relevant concentrations for a given exposure period; nematode motility is assessed by stimulating the nematode movement, where a lack of motility indicates that the compound is nematostatic. To distinguish whether the effect is nematostatic or nematicidal, the nematodes are incubated in distilled water for a recovery assessment; failure to recover qualifies the compound as nematicidal. A compound may be nematicidal or have a nematostatic effect depending on the dose of the compound and exposure time, as nematodes may recover at lower doses or die at higher doses [26]. The metabolites may lack nematostatic/nematicidal effects but may possess repellent activity which interferes with nematode chemoreceptors, hence impairing nematode host-finding abilities and causing mortality due to starvation. Nematode host-finding abilities are evaluated in a chemotaxis assay, where the movement of the nematode from a centre of inoculation, usually on an agar plate, is monitored and the metabolite is rated either as a strong/weak repellent or an attractant [32]. In other assays, seedlings of the plant are used to evaluate the attraction and repulsion, and this focuses mainly on the compounds being exuded by the roots [59]. The findings obtained in controlled laboratory conditions are sometimes not consistent with those observed in plants. Some of the factors contributing to the disparity are (i) some alkaloids from Epichloë are only produced in the plant, and (ii) the level concentrations of the metabolites from these interactions are greatly influenced by the host plant as the environment in which it grows in can alter the biosynthetic pathways involved in the production of the metabolites [17,61]. Alkaloids produced by grass–endophyte interactions have varying direct effects on nematode species depending on the grass–endophyte interactions involved, which determine which alkaloids are produced [9]. Factors such as the class of the alkaloid, concentration of the alkaloid, exposure time, part of the plant the extract is obtained, i.e., shoots/roots, and age of the plant have been shown to cause the variations in mortality, motility, and attraction/repulsion activity to the nematodes. In vitro bioassays primarily focus on the ability to kill, repel, or paralyse the nematodes, whereas other mechanisms maybe at play; therefore, there might be an underestimation or overestimation of the potential of the grass–endophyte interaction. However, these in vitro assays (Table 2) are an essential initial step to understanding the activity of fungal metabolites and enable the strategic screening of bioactive compounds for the development of nematode control strategies for field application.

Assays have been conducted using shoot and root extracts, exudates as well as purified forms of the alkaloids. Purified forms of loline, ergovaline, and α-ergocryptine have been mostly documented to possess nematicidal activity while ergonovine has mostly been associated with nematostatic activity [8]. Ergot alkaloids are known to have capabilities of acting as either stimulants or inhibitors at the receptors of the monoamine neurotransmitters serotonin, dopamine, adrenaline, and noradrenaline, and disruption of the central nervous system [43,62,63]. Nematicidal and nematostatic effects were reported in an experiment with different purified alkaloids towards *P. scribneri*. After 72 h exposure, ergovaline caused 100% loss in motility at the lowest dose of 5 µg mL^−1^ while loline and α-ergocryptine achieved 100% loss in motility at doses of 50 µg mL^−1^. Nematodes exposed to ergovaline did not recover after incubation in water (nematicidal effect), while nematodes recovered in water following ergonovine (5–100 µg mL^−1^), ergocornine (all concentrations), and loline (5–50 µg mL^−1^) treatments (nematostatic effect). Synergistic effects were also observed when the alkaloids were tested in combinations. A mixture of loline + α-ergocryptine + ergocornine caused 100% loss of motility after 72 h exposure across all concentrations tested (5–250 µg mL^−1^), and this was irreversible after rinsing the nematodes in distilled water. Loline + α-ergocryptine and loline + α-ergocornine caused a 97% decrease in motility at the 50–250 µg mL^−1^ concentration range and this was also an irreversible effect [32]. Purified ergot alkaloids, commonly observed in the interaction between perennial ryegrass infected with *Epichloë* spp., showed that not all ergot alkaloids are capable of immobilising *P. scribneri*. Various alkaloids including 5 µM ergovaline, 21 µM agroclavines, and a mixture of setoclavine and agroclavines at 7 µM and 34 µM, respectively, were tested. Only 5 µM ergovaline resulted in 50% reduced motility after 24 h when compared to the control solvent. No reduction in motility was observed for the agroclavines alkaloids and the mixture of agroclavines and its oxidised derivative setoclavine [45]; however, the experiment was not conclusive on the nematicidal aspect of the ergovaline as the nematodes were not incubated in water for a recovery assessment. The negative effect of metabolites associated with endophyte–grass interactions remained consistent for *P. scribneri*, even when crude extracts were tested. Root extracts of the tall fescue variety Jesup colonised (E+) verses non-colonised (E−) were compared in vitro. Methanolic root extracts of 22-week-old plants were tested against *P. scribinieri* at six concentrations ranging from 111.5 to 2400 µg mL^−1^. After a 72 h exposure period, the number of motile nematodes were significantly lower in E+ compared to E− plants across all concentrations. However, some nematodes recovered after incubation in distilled water, indicating that the root extracts had a nematostatic effect [64]. Apart from negatively affecting juveniles/motile stages of nematodes, crude extracts have been reported to directly affect nematode egg hatching. Root exudates, shoot extracts, and root extracts obtained from tall fescue Jesup (Max Q) colonised with *E. coenophialum* resulted in egg hatch inhibition of *M. incognita*. Seven days post-exposure, hatch inhibition values of 46% and 34% were achieved at the highest concentration of 100% for the shoot and root extracts, respectively. Juvenile activity was also reduced by 33% and 21% by day 7 in the root and shoot extracts, respectively, at the highest concentration. Root exudates also reduced egg hatching and juvenile activity with increasing exudate concentration; egg hatching and juvenile activity was reduced by 98% and 40%, respectively, by day 7 at the highest concentration of 1.4% [60]. Contrary results on hatching inhibition of *M. incognita* were obtained when using hybrid *Festulolium* spp. colonised with *Epichloë uncinata*; in this study, methanolic extracts obtained from glasshouse-grown plants did not affect the hatching. However, methanolic root and shoot extracts from different lines reduced juvenile activity. Most of the root extracts (except U6 E+ at 200 µg mL^−1^) had significantly reduced active J2 (12.7–18.3%) when compared to the controls on day 7. All shoot extracts resulted in significant decreases in J2 activity on day 7 (9.0–17.2%). The decreases were, however, not significant. In this study, the presence of the endophyte had no effect on the extract activity as both E+ and E− grasses affected juvenile activity [1]. A different approach was used in a study investigating the direct effect on juveniles of *M. incognita*; in the study, the endophyte fungi were isolated and the fungal filtrates obtained were exposed to the juveniles. Fungal filtrates were derived from different grass and fungal associations, i.e., cultivated grass *S. arundinaceus*–*E. coenophialum* and wild-grass *Leymus chinensis*–*Epichloë* spp., and wild grass *Achnatherum sibiricum*–*E. sibiricum*. The exposure time and concentration of the fungal filtrates had a significant effect on mortality. In *L. chiniensis*, the highest mortality was recorded after 72 h in undiluted fungal filtrate while in *N. sibiricum*, the mortality of the undiluted fungal filtrate was significantly higher after 24 and 48 h exposure periods compared to all other concentrations tested. The highest mortality in all the endophytes tested was recorded in the undiluted culture filtrates where they caused significantly higher J2 mortality (72.6% for *N. coenophialum*, 91.7% for *Epichloë* sp., and 66.8% for *N. sibiricum*), indicating that the fungal filtrates have a nematicidal effect. A comparison of juvenile root invasion of *M. incognita* post-treatment with fungal filtrates showed that *N. sibiricum* culture filtrates reduced penetration in cucumber roots by 45%. The culture filtrate of *Epichloë LC* also lowered the number of penetrations, although the difference was not statistically significant [59]. Repulsion and attraction of colonised verses non-colonised grasses have also been compared either using extracts or whole seedlings. In an assay investigating the attraction/repulsion activity of root extracts from tall fescue, the age and concentration of root extracts were shown to influence attraction and repulsion activity to *P. scribneri*. Tall fescue root extracts obtained from 0 to 30-day-old E+ and E− plants had a neutral effect, compared to 45-day-old plants, which showed a strong repellent effect at 100–400 µg mL^−1^, while lower repellence was recorded at 5–50 µg mL^−1^ for E+ plants. At day 60, concentrations of 50–400 µg mL^−1^ for E+ had a strong repellent effect while 5–20 µg mL^−1^ provided weak repellent effects. Root extracts of E− plants had an attractant effect at all concentrations [64]. The effect of alkaloid concentration in determining repulsion and attraction was also confirmed using purified alkaloids, where the chemotaxis factor (Cf) varied with concentration. The effects of ergovaline, ergotamine, ergonovine (ergot alkaloids), and N-formylloline (loline alkaloid) were evaluated in a chemotaxis assay. Ergovaline had a strong repellent effect (Cf = 0) at 100–200 µg mL^−1^ and a weak repellent effect from 1 to 50 µg mL^−1^, with the ability to cause mortality at 1–5 µg mL^−1^. Ergotamine was an attractant (Cf = 2.5–4) at all concentrations and the attraction led to nematode mortality. Ergonovine was a weak repellent (Cf = 0.2–0.4) at 50–200 µg mL^−1^ and an attractant at lower doses of 1–20 µg mL^−1^, and did not cause nematode mortality. N-formylloline (loline) and α-ergocryptine were weak repellents (Cf = 0.2–0.4) at high concentrations of 50–200 µg mL^−1^ and attractants (Cf = 2–3) at concentrations of 1–20 µg mL^−1^ [32]. A chemotaxis assay performed on agar plates using seedlings of *F. arundinaceus* colonised with *E. coenophialum* (E+) and non-colonised (E−) ones were assessed for their attraction/repulsion activity. A significantly greater proportion of *M. incognita* juveniles was on the E− side compared to the E+ side, whereas no difference was seen in agar plates without seedlings, showing the repellent effect of compounds released from the root system of E+ seedlings [59].

## 6. Host Status of Endophyte Grasses to Plant Parasitic Nematodes (PPNs)

The secondary metabolites produced by grass–endophyte interactions in plants can have negative effects to the PPNs either directly through the ingestion of toxins, hence causing mortality or repulsion which inhibits the nematode host-finding ability, resulting in death due to starvation [26]. Indirectly, the endophytes are capable of inducing host resistance to PPNs, which is one of the mechanisms also used by other antagonistic endophytes [18,65]. This induction primes the host plant’s response to nematode penetration and establishment, therefore preventing feeding and subsequent nematode development [9]. This involves the activation of genes responsible for producing a variety of phytohormones, phytoalexins, volatile organic compounds, pathogenesis-related proteins, and initiating the salicylic acid, jasmonic acid, and ethylene pathways, which serve to safeguard plants from stressors. Inoculation of the *Fusarium oxysporum* (one of the common antagonistic fungal endophytes against PPNs) endophyte on half of the banana roots in a split root experimental design, showed that the endophyte induced a systemic resistance where the uninoculated half of the root also supressed *Radopholus similis* [66]. Some of these defence mechanisms counteract stressors such as PPN, while others, such as phytohormones, promote plant growth and offset the damage caused by stressors [67]. In addition to induced host resistance, metabolic resistance is another scenario where the nematode may attempt to penetrate the host but encounters constitutively formed toxic metabolites which deter it from infecting the host [26]. Furthermore, endophytes can also influence the composition and production of root exudates, further inhibiting PPNs. This has been shown in *M. incognita* repulsion to root exudates extracted from roots colonized by *F. oxysporum* and preferring exudates from tomato [68]. Endophytes also deter PPNs by outcompeting them for resources [67,69]. For instance, *F. oxysporum* isolated from banana incapacitated and eliminated *Pratylenchus*, *Goodeyi* [70], while *Chaetomium globosum* produced secondary metabolites such as chaetoglobosin A, chaetoglobosin B, flavipin, 3-methoxyepicoccone, and 4,5,6-trihydroxy-7-methylphthalide, which had direct effects against *M. incognita* [71]. Various grass–endophyte associations have been investigated for their ability to suppress PPNs. Endophyte-colonised tall fescues have been shown to reduce the numbers of PPNs such as *Pratylenchus* spp. [32,64] and *Meloidogyne* spp. [1,72]. Despite the fact that the endophyte hyphae in grass–endophyte interactions do not occur in the root system, it has been proposed that the metabolites responsible for interacting with the nematodes in the roots are translocated from the leaves and stems which are the points of synthesis [2]. This was shown to be true as *Epichloe* spp. strains deficient in ergot alkaloid production were unable to reduce numbers of *Pratylenchus* spp. as compared to ergot-producing strains which had a negative effect [31]. In contrast, other studies showed that the concentrations of the translocated ergot alkaloids in the roots were very low. Knockout mutants having their ergot alkaloid biosynthesis pathway silenced were still able to effectively supress nematodes, hence concluding that the ergot alkaloids were not solely responsible for nematode suppression [45]. However, in other groups of endophytes associated with antinematode activity such as non-pathogenic strains of *F. oxysporum*, the culture filtrates have been shown to negatively affect *M. incognita* indicating that the direct interaction of endophyte toxins and nematodes can be a mechanism used by endophytes to suppress PPNs [3]. The mechanisms of action exhibited by the different grass–endophyte combinations therefore determine which nematodes can be negatively impacted as the different nematode lifestyles may render them either vulnerable to the toxins or tolerant [2]. For instance, endoparasitic and migratory endoparasitic nematodes, e.g., *Pratylenchus* spp. and *Meloidogyne* spp. which reside within the host tissue have longer exposure to the toxic compounds released whereas ectoparasitic nematodes feeding externally might be exposed for shorter periods as they migrate from one root hair to the other [73], which implies that the concentration of metabolites exuded must be high enough to effectively suppress ectoparasites within a short period of time [2]. The mechanisms of induced resistance by the endophyte in this case would be more suitable for targeting ectoparasitic nematodes. The concentration of the alkaloids in grass–endophyte interactions have been reported to elevate upon herbivore damage. The wounding causes immobilisation of the stored nutrients resulting in increased levels of alkaloids, which in turn induce a resistance response [8]. Following herbivore damage, the plant may develop chemical or structural changes that result in resistance. Structural changes in endophyte-colonised grasses such as thickening of the inner walls of endodermal cells has been previously reported in the endophyte-colonised tall fescue variety KY31 when compared to the E− control, and was concluded to be a resistance mechanism employed by KY31 [2]. This genotype has also been shown to have a degree of drought tolerance in comparison to non-colonised grass, and this tolerance correlates with the resistance observed to nematode infection (*P. scribneri* and *Tylenchorynchus acutus*) [74]. The chitinase activity of the tall fescue cv. KY31 was also shown to dramatically increase in the foliage upon inoculation with *M. marylandi* when compared to a less persistent tall fescue cultivar Johnstone. The study also showed that *Epichloë* spp.-colonised fescue had higher chitinase than endophyte-free plants. This suggests that the symbiosis between *Epichloë* spp. and KY31 grass, which is known for its resistance to pathogens and insects, may contribute to the increased chitinase level [75]. The variations in the multiplication of nematodes on different grass–endophyte combinations and non-colonised grasses are summarised in Table 3 below.

In some instances, the endophyte presence has significant effects on PPN reduction while in some cases, differences between E+ and E− have not been observed. While endophytes can affect the susceptibility of grasses to nematodes, host status has also been reported to be a major factor as some plant cultivars are non-hosts whether in the presence or absence of endophytes [1]. Host suitability studies of different grass–endophyte combinations have shown that differences in nematode suppression exist depending on the grass genotype involved, the grass–endophyte interaction, the endophyte strain, and also the nematode species in question [1,76]. A study conducted to test the host status of hybrid grass, *Festulolium* spp. with or without the fungal endophyte *E. uncinata*, showed that the Festulolium lines were poor hosts to *M. incognita* with or without the fungal endophyte [1]. Similar results were obtained in a study investigating the effect of Italian ryegrass colonised with *E. uncinatum* and non-colonised samples, on the reproduction of four agriculturally important PPNs: *M. incognita*, *M. arenaria*, *Pratylenchus coffeae*, and *P. penetrans*. The comparison of E+ and E− individuals revealed that colonisation with *E. uncinatum* had no effect on Italian ryegrasses’ host suitability to the four nematodes [77]. Under glasshouse conditions in pot experiments, a contrary result was reported for *P. scribneri*, where pots with E+ plants had 49 to 85 nematodes compared to 467 to 750 nematodes per pot for E− tall fescue 60 days post-inoculation. Endophyte colonisation was additionally seen to affect the fresh root weight where E+ plants had a higher weight compared to E− plants [64]. In another glasshouse study, the host status of tall fescue varieties was dependent on the nematode species; in this study, host suitability of *M. incognita*, *M. javanica*, *M. arenaria*, and *M. hapla* were tested on (i) wild-type Jesup (E+), (ii) Jesup (E−), (iii) Jesup Max-Q (E−), and (iv) Georgia 5 (E+), while peach (*Prunus persica* L.) was included as a known susceptible host. All tall fescue cultivars were classified as highly resistant to *M. incognita*. The cultivar Jesup Max-Q was rated as highly resistant to *M. incognita*, an excellent host to *M. javanica*, and a good host to *M. arenaria*, while Jesup wild-type was rated as a poor host to *M. incognita* but a good host to *M. javanica* and *M. arenaria*. A follow-up study was conducted to investigate which stage of *M. incognita* development was disrupted by Jesup (Max-Q). The mechanisms of resistance of Jesup (Max-Q) were shown to occur before and during root penetration with low juvenile root penetration and a failure of completing their life cycle. The number of juveniles penetrating in the control tomato roots were 3 to 7 times higher, had 40 to 80 times more females and egg masses, and 10 to 83 times more galls/plant than tomatoes grown after Jesup (Max-Q) [60].

When similar tall fescue varieties, i.e., cv. Jesup and cv. Georgia with two non-ergot-producing strains, AR542 and AR584, were compared against *Pratylenchus* spp. populations under glasshouse conditions, a contrary result was reported, where the presence of the endophyte significantly suppressed *Pratylenchus* spp. populations, while the non-ergot strains had no effect [31]; a similar result was reported for *Pratylenchus vulnus*, where results from the first experiment of the study showed that Jesup (E−) supported greater reproduction than Jesup (E+) and Jesup (Max-Q); however, in the second experiment, *P. vulnus* densities did not differ after cultivation on E+ or E− plants. In both tests, all tall fescue varieties were rated as poor hosts to *P. vulnus*, as compared to the control peach plants where they greatly multiplied [76]. In perennial ryegrass colonised with the endophyte *N. lolli*, *P. scribneri* was still suppressed, even after knockout of the genes responsible for ergot alkaloid production. Knockout of *dmaW* eliminated all ergot alkaloids whereas knockout of *lpsA* allowed the accumulation of clavine alkaloids and lysergic acid but not of ergovaline or lysergic acid amides. Endophyte status had a significant effect on *P. scribneri* densities, where all treatments with E+ had lower densities compared to E−. The ergot alkaloid pathway status had no effect on nematode suppression indicating that ergot alkaloid-free endophytes can supress this nematode. This indicates that mutants lacking ergot alkaloids have other mechanisms that aid in nematode suppression [45]. The lack of significance of endophyte presence on the host status of grasses against ectoparasitic nematodes has been consistent in different studies. Ectoparasitic nematodes are known to feed externally without penetrating the roots and migrate from one root hair to another. As such, it has been shown that their feeding behaviour might enable them to evade the effect of the metabolites produced by the grass–endophyte interactions [2]. Tests carried out on four tall fescue cultivars, i.e., (i) wild-type Jesup (E+), (ii) Jesup (E−), (iii) Jesup Max-Q (E−), and (iv) Greece 5′ (E+) against *Mesocriconema xenoplax* showed that all the varieties were rated as good hosts despite some being colonised by an endophyte [76]. Similar results were obtained in glasshouse studies conducted in New Zealand investigating the host suitability of 15 common pasture plants to the ectoparasitic nematode *Helicotylenchus pseudorobustus*; it was found that tall fescue associated with its fungal endophyte *N. lolii* was an excellent host to *H. pseudorobustus* with a high juvenile to female ratio. Even in the absence of the host, *H. pseudorobustus* was found to persist in the soil. Other grasses and clovers were good hosts while Caucasian clover, subterranean clover, plantain, and yellow yarrow were classified as maintenance hosts. Additionally, the number of free-living nematodes (bacterivores) were also seen to significantly increase in response to increases in *H. pseudorobustus* densities [79]. In a similar study investigating the nematode host range of common pastures in New Zealand, perennial ryegrass with and without the endophyte *Epichloë* sp. was also classified as a good host to the ectoparasitic nematodes *Paratylenchus nanus* and *Paratrichodorus minor* [80]. Results from a 6-month glasshouse experiment comparing tall fescue varieties adapted to different environmental conditions, continental cultivars, Kentucky 31 (Ky31+), Texoma MaxQII (TMQ+), the Mediterranean cultivar Flecha MaxQ (FL+), and their respective non-colonised controls, against a mixed population of lesion, spiral, ring, and stunt nematodes, also showed that as a main effect, the endophyte status had no effect on the densities of ring, stunt, and spiral nematodes. The endophyte and cultivar combination had an effect on the spiral and stunt nematodes where the FL+ had significantly higher spiral and stunt nematodes compared to other cultivar and endophyte combinations. The cultivar FL+ was rated as a good host for spiral, stunt, and ring nematodes, while Ky31+, TMQ+, and TMQ− were rated as poor hosts for stunt, ring, and spiral nematodes. Ky31− was rated as a poor host for stunt and ring nematodes, but a good host for spiral nematodes. The endophyte status was also shown to have no effect on lesion nematodes; however, the results for lesion nematodes were considered inconclusive as the study did not examine the densities within the roots as being migratory endoparasites, they could be in both the roots and the soil [78].

Under field conditions, efficacy trials have shown that some of the varieties are able to supress PPNs. The resurgence of *Meloidogyne* spp. in soil previously planted with the tall fescue varieties and the susceptible check, peach was investigated by planting tomato plants. In this trial, Jesup-Max-Q supressed the resurgence of *M. incognita* and *M. hapla* but not *M. javanica* and *M. arenaria*, indicating that it could be used as a pre-plant strategy for the management of *M. incognita* and *M. hapla* [72]. The same variety (Jesup-Max-Q) was tested under field conditions as a pre-plant alternative to chemical nematicides on *M. incognita* populations prior to peach orchard establishment over a period of 7 years. The study compared (i) 1 year of peach followed by 1 year of Jesup (Max-Q), (ii) 2 years of continuous Jesup (Max-Q), (iii) 2 years of continuous peach, and (iv) 2 years of continuous peach followed by fumigation with 1,3-dichloropropene (1,3-D). The pre-plant treatments did not have a significant impact on nematode population density at first, but later sampling dates (13 months after planting) revealed lower populations in plots planted with grass and Jesup (Max-Q) than continuous peach plots. Over a three-year period, non-fumigated plots had the highest nematode populations while fumigated plots had the lowest. Tree growth was the greatest in fumigated and Jesup (Max-Q) plots, mediocre in grass-planted plots, and minimal in non-fumigated plots, according to trunk diameter measurements. Overall, the findings suggested that pre-planting and post-planting treatments had an impact on nematode populations and tree growth in peach orchards [81]. The rate at which the endophyte colonised the tillers was shown to undermine the efficacy of the endophyte in nematode suppression under field conditions. A comparison of ryegrass to tall fescue E+ and E− showed that there were seven times more *Pratylenchus* spp. in ryegrass compared to tall fescue. The endophyte status did not significantly affect the densities of *Pratylenchus* spp., *H. pseudorobustus*, and *Tylenchus* spp., but the total nematode numbers were 26% lower under endophyte-colonised grass than the non-colonised grass, indicating that the endophyte strain was unstable, hence resulting in a lower suppression which would have been higher if all tillers were colonised by the endophyte [82].

## 7. Conclusions and Future Perspectives

The susceptibility or resistance of grass–endophyte associations to nematodes cannot be generalized, and it is necessary to evaluate individual grass–endophyte combinations to determine their susceptibility to each nematode species. The symbiotic interaction between the grass and endophyte is highly complex and exhibits significant variability, presenting both advantages and disadvantages. The advantage is the potential identification of combinations from the diversity for further exploration in nematode suppression, while the disadvantage lies in the excessive uncontrolled variation encountered at the field scale. The research on grass–endophyte interactions has primarily focused on greenhouse and in vitro assays, with very few documented field trials on the efficacy of these interactions. It is evident that grass–endophyte interactions may involve multiple mechanisms with regard to nematode suppression. In vitro assays demonstrate that the extracts or purified compounds obtained from these interactions can have repellent effects, inhibit hatching, and directly cause mortality to the nematodes, indicating the sensitivity of some nematode species to the metabolites associated with these interactions. Glasshouse studies have also identified outstanding genotypes of tall fescue that have adapted to various environmental conditions, potentially affecting their ability to suppress nematodes. Research focusing on identifying fungal endophytes that maximize host defences could enhance the efficacy of nematode suppression. It is also necessary to select standardized grass–endophyte combinations, where the genotype and fungal strains can be used as models under defined conditions for comparative studies to identify the alkaloids produced, concentrations required, and determine which nematodes are sensitive to specific alkaloids. Such studies help elucidate the conditions and combinations of grasses and endophytes that could be exploited to provide greater efficacy in nematode suppression. The availability of these alkaloids in required concentrations in the root tissues and their potential exudation in the soil environment in different grass–endophyte interactions is a significant research gap that requires investigation, as it has direct implications on nematode suppression, especially for nematodes that feed in an ectoparasitic manner. The factors that affect the sensitivity of different nematodes to the different alkaloids need investigation, using in vitro and glasshouse-controlled conditions, which can serve as an important initial screening before implementation in field trials. Although Epichloë endophytes are not naturally found in modern cereal grasses, it has been demonstrated that they can be artificially inoculated into wheat, barley, and rye [83]. Artificially inoculated rye has been shown to suppress the prevalence of leaf streak (*Cercosporidium graminis*) and leaf rust (*Puccinia recondita*) [84], indicating the potential for exploration in nematode management.

## Figures and Tables

**Table 1:** Concentration of alkaloids in grass–endophyte interactions in different studies.

Grass Host	Endophyte	Grass Genotype	Alkaloids ^a^				Plant Tissue	Reference
			Ergopeptines	Lolines	Lolitrems	Peramine		
*Festuca arundinacea*	*E. coenophialum*		0.5	1100	0	2	Shoots	[53]
	*N. lolli*		1.2	0	23	18	Shoots	
*F. arundinacea*	*E. coenophialum*	*KY31*	1.72–6.81	2407–3427	−	−	Shoots	[30]
*F. arundinacea*	*A. coenophialum*		−	1544	−	−	Shoots	[53]
*Lolium perenne*	*E. lolii*		−	0	−	−		
	*E. starri*		−	0	−	−		
	*E. coenophialum*		−	1109	−	−		
	*E. typhina*		−	0	−	−		
	*E. lolli*		1.3	0	4.7	19		
	*E. coenophialum*		2.5	1000	0	29		
	*E. typhina*		0	0	0	53		
	*E. lolli X E. typhina*		4.8	0	0.4	22		
*Festulolium* spp.	*E. uncinata*	*U2*	−	358	−	−	Roots	[1]
		*U5*	−	270	−	−		
		*U6*	−	596	−	−		
		*U8*	−	590	−	−		
		*U10*	−	548	−	−		
*F. pratensis*	*E. uncinatum*	*FP53*	−	102	−	−	Roots	[39]
		*Fp246*	−	86	−	−	Roots	
		*Fp248*	−	123	−	−	Roots	
		*Fp408*	−	1444	−	−	Roots	
		*Fp87*	−	1334	−	−	Roots	
		*Fp358*	−	1251	−	−	Roots	
		*Fp391*	−	1368	−	−	Roots	
		*Fp345*	−	1474	−	−	Roots	
		*Fp262*	−	1330	−	−	Roots	
		*Fp440*	−	1725	−	−	Roots	
		*Fp390*	−	1362	−	−	Roots	
		*Fp430*	−	1320	−	−	Roots	
*F. pratensis*	*E. uncinatum*	*FP53*	−	38	−	−	Crown	[39]
		*Fp246*	−	45	−	−	Crown	
		*Fp248*	−	51	−	−	Crown	
		*Fp408*	−	1944	−	−	Crown	
		*Fp87*	−	2766	−	−	Crown	
		*Fp358*	−	1498	−	−	Crown	
		*Fp391*	−	1007	−	−	Crown	
		*Fp345*	−	1976	−	−	Crown	
		*Fp262*	−	1862	−	−	Crown	
		*Fp440*	−	2281	−	−	Crown	
		*Fp390*	−	1574	−	−	Crown	
		*Fp430*	−	1393	−	−	Crown	
		*FP53*	−	40	−	−	Shoots	
		*Fp246*	−	47	−	−	Shoots	
		*Fp248*	−	38	−	−	Shoots	
		*Fp408*	−	1374	−	−	Shoots	
*F. pratensis*	*E. uncinatum*	*Fp87*	−	1513	−	−	Shoots	[39]
		*Fp358*	−	907	−	−	Shoots	
		*Fp391*	−	772	−	−	Shoots	
		*Fp345*	−	1272	−	−	Shoots	
		*Fp262*	−	1297	−	−	Shoots	
		*Fp440*	−	1528	−	−	Shoots	
		*Fp390*	−	1251	−	−	Shoots	
		*Fp430*	−	840	−	−	Shoots	
*F. pratensis Huds*	*E. uncinatum*		−	670	−	−	Shoots	[54]
			−	1240	−	−	Shoots	
			−	610	−	−	Shoots	
			−	1160	−	−	Shoots	
			−	760	−	−	Shoots	
			−	500	−	−	Shoots	
			−	3180	−	−	Stem	
			−	4080	−	−	Stem	
			−	2600	−	−	Stem	
			−	2690	−	−	Stem	
			−	2600	−	−	Stem	
			−	1670	−	−	Stem	

^a^ Concentration of different classes of alkaloids in µg g^−1^ dry weight (DW)**;** − Not analysed.

**Table 2:** Summary of in vitro tests evaluating the direct effects of alkaloids from grass–endophyte interactions on different nematode species.

Nematode Species	Grass Genotype	Endophyte Species/Alkaloids Tested	Exposure Material	Assay	Nematode Stage	Dose	Exposure Time	Effect	%Efficacy	Reference
*Meloidogyne incognita*	*S. arundinacea*	*Epichloë coenophialum*	Seedlings	Chemotaxis	Juveniles		2 h	Repulsion	Chemotaxis factor = 0	[59]
*M.incognita*	*S. arundinacea*	*E. coenophialum*	Fungal filtrate	Mortality	Juveniles	100%	72 h	Nematicidal	72%	[59]
	*Leymus chiniensis*	*Epichloë* sp.	Fungal filtrate		Juveniles	100% fungal filtrate	72 h		91.7%	
	*Achnatherum sibiricum*	*E. sibiricum*	Fungal filtrate		Juveniles	100% fungal filtrate	72 h		66.8%	
*Pratylenchus scribinieri*		Ergovaline	Purified alkaloids	Mortality	Juveniles	5 µg mL^−1^	72 h	Nematicidal	100%	[32]
		Lolines				50 µg mL^−1^	72 h	Nematostatic	100%	
		Ergocryptine				50 µg mL^−1^	72 h	Nematostatic	100%	
*P. scribinieri*	*Festuca arundinacea*	*E. coenophialum*	Root extracts	Mortality	Juveniles	2400 µg mL^−^	72 h	Nematostatic	80%	[32]
*P. scribinieri*		Ergovaline	Purified alkaloids	Mortality	Juveniles	5 µM	24 h	Nematicidal	50%	[45]
		Agroclavine				21 µM	24 h	No effect		
		Setoclavine + Agroclavine				7 µM + 34 µM	24 h	No effect		
*M. incognita*	*Festuca arundinacea*	*E. coenophialum*	Root exudates	Mortality	Juveniles	1.4 *w*/*w*	7 days	Nematostatic	39.5%	[60]
					Eggs	1.4 *w*/*w*	7 days	Hatching inhibition	97.6%	
			Root extracts		Juveniles	100%	7 days	Nematostatic	32%	
					Eggs	100%	7 days	Hatching inhibition	34%	
			Shoot extracts		Juveniles	100%	7 days	Nematostatic	21%	
					Eggs	100%	7 days	Hatching inhibition	46%	
*P. scribinieri*	*Festuca arundinacea*	*E. coenophialum*	Root extracts	Chemotaxis	Juveniles	100–400 µg mL^−1^	2 h	Strong repellent	Chemotaxis factor = 0	[32]
*P. scribinieri*		Ergovaline	Purified alkaloids	Chemotaxis	Juveniles	100–200 µg mL^−1^	2 h	Strong repellent	Chemotaxis factor = 0	[32]
		Ergotamine				50–200 µg mL^−1^		Attractant	Cf = 2–3	
		Ergonovine				50–200 µg mL^−1^		Weak repellent	Cf = 0.2–0.4	
		N-Formylloline				50–200 µg mL^−1^		Weak repellent	Cf = 0.2–0.4	
*M. incognita*	*Festulolium* spp.	*Epichloë uncinata*	Root extracts	Mortality	Juveniles			Nematicidal	12.7–18.3%	[1]
			Shoot extracts					Nematicidal	9–19.2%	

**Table 3:** Summary of pot experiments on the multiplication of different nematode species on colonised and non-colonised grass genotypes.

Nematode Species	Endophyte		Grass		Reproduction on Colonised (E+) or Non-Colonised (E−)						
	Species	Strain	Genotype	Cultivar/Variety	Initial Densities (Pi)/Pot	Final Densities (Pf)		Assessment	Trial Duration (Days)	Country	References
						E+	E−				
*Meloidogyne incognita*	*Epichloë uncinate*	U6 U8 U10	Festulolium hybrids	FHCDO802 BUS 10–12 FHAB0802 ABA 10–22 FHCD0802 BUS 10–13	5000	285.5 71.2 803.2	500 NS 63.1 NS 95.3 NS	Eggs/gram roots	49	USA	[1]
*Pratylenchus scribinieri*	*Epichloë coenophialum*		*Festuca arundinacea*	Jesup.	1500	75 1	600 * 1734 *	Nematodes/100 cm^3^ soil Nematodes/gram roots	40–45	USA	[64]
*Pratylenchus* spp.	*E. coenophialum*	Endemic		Georgia Jesup.	984	20–30 50–70	150–190 * 130–140 *	Nematodes in roots/pot	56	USA	[31]
*P. vulnus* *Mesocriconema xenoplax*	*E. coenophialum*		*F. pratensis*	Wild-type Jesup Jesup (Max-Q) Georgia Wild-type Jesup Jesup (Max-Q Georgia	3000 1000	2 0 6 8 6 6	12 * 12 * 12 NS 17 NS 17 NS 17 NS	Nematodes in 100 cm^3^ soil	153 159	USA	[76]
*M.incognita* *M.arenaria* *P. coffeae* *P. penetrans*	*N. uncinatum*		*Lolium multiflorum-rum*	Bishanon JFIR-18 Bishanon JFIR-18 Bishanon JFIR-18 Bishanon JFIR-18	500 500 300 300	50.5 37 41 66.2 721.50 288.2 412.40 367.10	42.5 NS 44 NS 39 NS 57.4 NS 515 NS 291.4 NS 501.6 NS 370.1 NS	Egg mass/root system Nematodes/root system	42 48	Japan	[77]
*M.incognita*			*F. arundinacea*	Wild-type Jesup Jesup (Max-Q) Georgia 5 Bulldog 51	3000	0 0 0 7	15 NS 15 NS 15 NS 15 NS	Eggs/gram root		USA	[72]
*Tylenchorynchus* spp. *Criconemella* spp. *Helicotylenchus* spp.				Kentucky 31 Texoma MaxQII Flecha MaxQ Kentucky 31 Texoma MaxQII Flecha MaxQ Kentucky 31 Texoma MaxQII Flecha MaxQ Kentucky 31 Texoma MaxQII Flecha MaxQ	270 High rate (800) Low rate (250) 225	20 35 91 6 159 606 14 34 1026 55 84 330	40 NS 32 NS 40 * 246 NS 236 NS 311 NS 64 NS 291 NS 162 * 174 NS 80 NS 147 *	Nematodes in 100 cm^3^ soil	180	USA	[78]
*M.incognita*	*E sibiricum* *E. coenophialum*		*Achnatherum sibiricum* *F.arundin-acea*	Wild-type Kentucky 31	1000	10 0–5	20–25 * 10–20 *	Nematodes/root system	15	China	[59]
*P.scribneri*	*Epichloë* spp.	Wild-type Isolate Lp1	*Lolium perenne*	Isolate Lp1 lpsA knockout dmaW knockout	1000	80–100 100–150 100–110	400–410 * 400–410 * 400–410 *	Nematodes/pot	48	USA	[45]

*, statistically significant (*p* < 0.05); NS, not statistically significant (*p* > 0.05).

## Data Availability

No new data were created or analyzed in this study. Data sharing is not applicable to this article.
